# Differential transendothelial transport of adiponectin complexes

**DOI:** 10.1186/1475-2840-13-47

**Published:** 2014-02-20

**Authors:** Joseph M Rutkowski, Nils Halberg, Qiong A Wang, William L Holland, Jonathan Y Xia, Philipp E Scherer

**Affiliations:** 1 Touchstone Diabetes Center, Department of Internal Medicine, University of Texas Southwestern Medical Center, 5323 Harry Hines Boulevard, Dallas, TX 75390, USA; 2 Department of Cell Biology, University of Texas Southwestern Medical Center, 5323 Harry Hines Boulevard, Dallas, TX 75390, USA

**Keywords:** Adiponectin, Oligomerization, Endothelium, Ceramide, Exercise

## Abstract

**Background:**

Adiponectin’s effects on systemic physiology and cell-specific responses are well-defined, but little is known about how this insulin-sensitizing and anti-inflammatory adipokine reaches its target cells. All molecules face active and passive transport limitations, but adiponectin is particularly noteworthy due to the diverse size range and high molecular weights of its oligomers. Additionally, its metabolic target organs possess a range of endothelial permeability.

**Methods:**

Full-length recombinant murine adiponectin was produced and oligomer fractions isolated by gel filtration. Adiponectin complex sizes were measured by dynamic light scattering to determine Stokes radii. Transendothelial transport of purified oligomers was quantitatively assessed under a number of different conditions *in vitro* using murine endothelial cells and *in vivo* using several mouse models of altered endothelial function.

**Results:**

Adiponectin oligomers exhibit large transport radii that limit transendothelial transport. Oligomerization is a significant determinant of flux across endothelial monolayers *in vitro*; low molecular weight adiponectin is preferentially transported. *In vivo* sampled sera from the heart, liver, and tail vein demonstrated significantly different complex distribution of lower molecular weight oligomers. Pharmacological interventions, such as PPARγ agonist treatment, differentially affect adiponectin plasma clearance and tissue uptake. Exercise induces enhanced adiponectin uptake to oxidative skeletal muscles, wherein adiponectin potently lowers ceramide levels. In total, endothelial barriers control adiponectin transport in a cell- and tissue-specific manner.

**Conclusions:**

Adiponectin oligomer efficacy in a given tissue may therefore be endothelial transport mediated. Targeting endothelial dysfunction in the metabolic syndrome through exercise and pharmaceuticals may afford an effective approach to increasing adiponectin’s beneficial effects.

## Background

With the alarming rise of obesity and obesity-related diseases, understanding the endocrine functions of adipose tissue and adipocyte-secreted adipokines in regulating systemic metabolism and insulin sensitivity has become critical. Inversely expressed with obesity, the adipokine adiponectin has overall positive insulin-sensitizing and metabolic effects via signaling in various tissues such as liver and skeletal muscle. Adiponectin is unique in its post-translational modifications that result in secreted oligomers of 90 kDa trimers, 180 kDa hexamers (LMW) and 18- and potentially 36-mers in found in circulation [1,2]. Our laboratory has demonstrated differential efficacy of various sized adiponectin complexes on systemic insulin sensitivity with the high molecular weight (HMW) oligomers, or HMW:Trimer ratio, correlating best with insulin sensitivity in both mouse and human studies [2-6].

Tranport of large proteins, lipoproteins, and chylomicrons occurs through open fenestrations (100–200 nm pores) across filter or secretory tissue endothelium (e.g., liver or adipose tissue) [7]. Other tissues, such as healthy lung, heart and skeletal muscle, lack fenestrae and thus have ‘tighter’ endothelial transport barriers [8]. Under healthy conditions in these blood vessels, paracellular endothelial transport is limited to small solutes (radii <3 nm). The transport of molecules with Stokes radii greater than approximately 3 nm across continuous endothelia, such as the tight endothelial barriers in skeletal muscle, requires active transport through mechanisms ranging from caveolar vesicular trafficking to the formation of transient transendothelial pores [9]. The endothelium is also a plastic transport barrier, as an array of cytokines and growth factors alter barrier function through modulating endothelial nitric oxide synthesis, cell-cell junctions, and trans-cellular trafficking mechanisms [10-13]. With endothelial dysfunction in obesity arising from altered insulin signaling or fatty acid-induced inflammation, these transport mechamisms are compromised [10,13,14]. Changes in endothelial function and hormone transport thus, in turn, affect tissue and whole body metabolic function. For example, transendothelial transport is a necessary step in insulin uptake to skeletal muscle despite insulin’s small size compared to other hormonal factors [15]; genetic manipulations that result in altered vascular endothelial permeability alter insulin uptake and action [16,17]. Due to the potential transport limitations of adiponectin, in particlular its highly beneficial HMW form, we propose that the endothelium presents a transport barrier to adiponectin that significantly affects its access to target cells.

We have previously demonstrated that the clearance time of adiponectin from the circulation is dependent on oligomer size [18]; metabolic dysfunction induced by high fat diet or the ob/ob mutation significantly reduced clearance. To quantify the transport limitations of adiponectin across endothelium, we produced full-length recombinant murine adiponectin and isolated from this purified HMW, LMW, and trimeric oligomer preparations. We calculate the transport parameters of each oligomer *in vitro* using dynamic light scattering and murine endothelial cell monolayers. *In vivo*, we quantify the effects of nitroglycerine and PPARγ-agonist treatment on adiponectin oligomer circulatory half-life and tissue fate. We find that exercise significantly increases adiponectin uptake and action in oxidative skeletal muscle. The non-trivial transendothelial transport of adiponectin oligomers is therefore an important and potentially rate-limiting factor for adiponectin to mediate its positive systemic effects.

## Methods

### Protein production and labeling

Several milligrams of full-length murine adiponectin was produced in stably transfected HEK 293 T cells as previously described to generate a mix of HMW, LMW, and trimeric forms [5]. Adiponectin was repeatedly fractionated using an ÁKTA FPLC system (GE Healthcare Bio-Sciences Corp., Piscataway, NJ) and Superdex 200 10/300 GL column (GE Healthcare), 200 μg per load, into 250 μL fractions in 20 mM Tris buffer (pH?=?8.0) [2]. Adiponectin oligomer distribution was determined by immunoblotting of the first 24 fractions as previously described [19]. Briefly, 4 μL of dithiothreitol containing Laemmli buffer was added to 20 μL of the first 24 fractions and the samples were heated at 95?ºC for 5 minutes. 20 μL of the mixture was loaded onto 26-well Bio-Rad Criterion XT Bis-Tris gels (4-12%) (Hercules, CA), separated by electrophoresis, transferred to nitrocellulose membranes and immunoblotted with a rabbit polyclonal sera against murine adiponectin [2]. Membranes were scanned on an LI-COR Odyssey infrared imaging system following secondary labeling with a goat anti-rabbit IR800 antibody (LI-COR).

Eluted fractions, designated by valleys in fraction immunoblot intensity, containing each oligomer were combined to obtain pure HMW, LMW, and trimeric preparations and concentrated to 1.0 mg/mL. [The exact fraction numbers pooled varied by preparation; pools were normalized batch-to-batch by collecting each species peak as identified by immunoblot intensity]. The purity of each final oligomer preparation was assessed by repeating the fractionation for immunoblotting. The percent of each species represented was calculated as sum of raw intensities for the given species fractions over the total intensities normalized to the peak value, as measured in the Odyssey software. Intensities are graphed as smoothed data (0^th^ order, 2-neighbor smoothing) in Prism software (GraphPad Software, Inc.).

Full-length preparations and individual oligomer species were used *in vitro*; HMW and LMW preps were used *in vivo*. Globular murine adiponectin (gAPN) (lacking adiponectin’s collagenous domain; 16.6 kDa) was purchased for comparison (PeproTech, Rocky Hill, NJ). Mouse IgGs were purchased from Sigma-Aldrich (St. Louis, MO).

HMW, LMW, and gAPN were labeled with LI-COR (Lincoln, NE) IRDye 800CW and IgGs labeled with IRDye 700DX (all at 1:1 molar ratios) according to the manufacturer’s protocol. 3 kDa Alexa594 dextran (Invitrogen, Carlsbad, CA) was used as a permeability control throughout.

### Adiponectin Oligomer Physical Size Measurements

Full-length recombinant adiponectin and the prepared fractions were measured by dynamic light scattering (DLS) on a temperature controlled Protein Solutions DynaPro Molecular Sizer. Dextrans, mouse IgG, and BSA were also measured for standards. Measurements were made at 25°C. Sizes were calculated in Dynamics V6.3.40 software after adjusting the baseline cutoffs such that the regularization fit matched the intensity auto correlations. A linear polysaccharide estimation was applied to dextrans and globular protein estimation used for the proteins. The three predominant species observed in measuring the full-length preparation were the focus for subsequent purified fraction positioning – and then only that peak fit for correlation precision. Multiple values were accepted and averaged with small changes in the upper baseline cutoff. All solutions were thrice measured at concentrations of approximately 10 μg/mL. Presented values are measured averages of three separate production preparations.

### Animals

Male FVB mice, aged 12–16 weeks were utilized for assessing adiponectin circulatory clearance. All mice were maintained with *ad libitum* access to water and standard chow throughout each experimental period. Age and weight-matched pairs were grouped for each injection (averaging 28-30 g). Caveolin-1 knockout mice were used on a fully backcrossed FVB background and compared to wild type matched litters [20]. Animal protocols were approved by the Institutional Animal Care and Use Committee at UT Southwestern Medical Center.

### Experimental treatments

Mice in the PPARγ agonist group were provided powdered standard chow diet containing 0.5 μg/g of the non-thiazolidinedione (nTZD) PPARγ agonist compound COOH [2-(2-(4-phenoxy-2-propylphenoxy)ethyl)indole-5-acetic acid] (a kind gift from Merck & Co., Inc. [21,22]) for one week prior to circulatory studies. Control mice also received powdered chow. For acute nitroglycerine circulatory studies, mice were gavaged 30 minutes prior to adiponectin injection with 1 mg/kg isosorbide dinitrate (CorePharma, Middlesex, NJ) [23,24]. Ground tablets were prepped in 2% Tween 80 in water (approximately 50 μL/gavage) with gavages repeatedly hourly. Control mice received equivalent volumes of 2% Tween 80 in water. Caveolin-1 knockout mice, previously described to exhibit increased paracellular albumin flux [25], were used without manipulation and compared to wildtype matched litters.

### *In vitro* endothelial permeability assay

Model murine endothelial cells lines, MS1, bEnd.3 (Bend), and EOMA, were acquired from ATCC (Manassas, VA) and maintained in DMEM (4 mmol/L L-glutamine, 4500 mg/L glucose, 1 mmol/L sodium pyruvate, and 1500 mg/L sodium bicarbonate) supplemented with 10% FBS and 0.5% pen strep (all from Thermo Scientific HyClone, Logna, UT). All cells were used at passages 3–5 from the supplier. Endothelial cells were seeded at a high density in transwell inserts (Corning, Tewksbury, MA) sized for 12-well plates. Polycarbonate membrane inserts were used for transport studies and polyester inserts were used for imaging, all with 3.0 μm pore sizes. Dextran flux was assessed to determine the experimental timeframe of 6 hours, in agreement with previous work with large molecule transport [26]. 6 hours prior to the experiment, cell medium was changed to 2% FBS. 10 μg/mL adiponectin and dextran were applied above the monolayer (luminal side) to the top well. After 6 hours, top (that above the monolayer) and bottom (below the cells and membrane) media were assessed for adiponectin and dextran concentrations by gel electrophoresis (as above) and fluorescence intensity measurements, respectively, and concentrations calculated by comparison to standard curves prepared in culture medium. Dextran fluorescence was measured on a POLARStar OPTIMA fluorescence plate reader (BMG LABTECH GmbH, Ortenberg, Germany). Permeability was calculated as previously detailed [26] using the known starting top concentrations and quantified bottom species concentrations. For dynamin inhibition 20 μL of 80 μmol/L Dynasore (Sigma) in DMSO was applied to the lower well 30 minutes prior to adiponectin (DMSO used as controls). The adiponectin oligomer distribution when full-form adiponectin was applied was assessed in the top and bottom media after 6 hours described as above. Cell-cell junctions were identified by an anti-mouse VE-Cadherin antibody (R&D Systems, Minneapolis, MN) labeled by an Alexafluor488-conjugated donkey anti-goat secondary antibody (Life Technologies). Fluorescence and IR800-labeled adiponectin were imaged on a Leica TCS SPF confocal microscope. At the end of experiments, RNA was isolated from cell monolayers using the Qiagen RNeasy Mini kit according the manufacturer’s instructions (Qiagen GmbH, Hilden, Germany). cDNA was synthesized using the iScript cDNA Synthesis Kit (Bio-Rad). Expression was quantified on an Applied Biosystems 7900 lightcycler using primers as listed in the Additional file 1: Table S1. Results are expressed relative to basal MS1 cell levels.

### Circulatory half-life quantifications

Endogenous tissue venous oligomer distributions were determined in sera collected from the tail vein, portal vein, and left ventricle under anesthesia using gel filtration as above and as described previously [2]. The clearance of exogenous HMW and LMW adiponectin were determined by quantifying the concentration of IR800-labeled injected adiponectin as previously described [18]: 0.05 μg/g adiponectin, IR700-labeled mouse IgG, and dextran were injected intravenously via the tail vein. Approximately 10 μL of blood was sampled from the tail tip into heparinized tubes at each time point for up to 180 minutes. DTT-denatured sera was separated by gel electrophoresis on Bio-Rad Criterion XT Bis-Tris gels (4-12%) for each time point. Gels were immediately scanned on an LI-COR Odyssey infrared imaging system to directly visualize and quantify the 32 kDa and 25 kDa adiponectin and IgG light-chain bands, respectively. Gels were transferred to nitrocellulose membranes and labeled for total murine IgG light-chain with a LI-COR goat anti-mouse IR700 antibody (1:5000) for total serum loading. Additionally, serum dextran fluorescence was scanned on a POLARStar OPTIMA fluorescence plate reader; 2 μL sera were added to 48 μL TBS to ensure the well bottoms were covered. Half-lives were calculated using a non-linear regression (decay) analysis of adiponectin:IgG ratios in Prism software.

At 180 minutes, mice were perfused through the left ventricle with 20 mL PBS under while under isofluorane anesthesia and sacrificed. Tissues were excised and immediately flash frozen. Tissues were scanned on the Odyssey imaging system to discern changes in adiponectin localization before homogenization in RIPA buffer. Homogenated protein concentrations were measured by BCA assay according to the manufacturer’s protocol (Thermo Scientific) and normalized across equivalent tissues (i.e., liver to liver or brain to brain). Following normalization, 2 μL of homogenate was dot blotted in a nitrocellulose membrane and scanned on the Odyssey. Tissue results are expressed as adiponectin:IgG ratios.

### Exercise studies

10 wildtype mice were exercise trained at low impact (<10 m/min) on a Exer-6 M treadmill (Columbus Instruments) following acclimatization every other day for one week to increase treadmill time from 1 hour to 2 hours. For the experiment, mice we i.v. injected with 0.05 μg/g IR800-labeled HMW adiponectin or saline vehicle and run at 16 m/min for 2 hours. Mice were sacrificed and a fresh set of skeletal muscles were scanned on a LI-COR Odyssey for IR800 adiponectin. The other set of muscles were flash frozen and later measured for ceramide content. Triceps and quadriceps represent glycolytic muscles while gastrocnemius (whole; a mix of fiber types) and soleus are oxidative.

### Ceramide content

Total ceramide content was quantified from lipid extracts of exercised skeletal muscles analyzed by LC/MS with a TSQ Quantum Ultra-triple quadrupole mass spectrometer outfitted with an electrospray ionization probe as described previously [27]. Ceramide content is reported as summation of C14:0-C24:0 ceramide species on a per muscle tissue mass basis.

### Statistical analysis

Transport and circulatory data were analyzed by two-way analysis of variance (ANOVA) using GraphPad Prism software. For *in vitro* analyses, oligomer size and cell type were used as variables. For circulation quantifications, time and treatment were used as variables; tissue and treatment were used as variables for tissue uptake. Bonferroni post-hoc analyses of multiple comparisons were used for all data. Differences in gene expression and tissue data were analyzed by two-tailed student’s t-test with unequal variance. Circulatory half-life determinations were calculated by non-linear regression analysis assuming an exponential one factor decay model in Prism. Transwell data are presented as box and whisker plots with error bars representing the 95% confidence interval. Tissue data are presented as means?±?SD, while circulation graphs present SE for graph visual clarity. Significances are indicated as *0.05 > *p* > 0.01, **0.01 > *p* > 0.001, ***p < 0.001 for adiponectin oligomer differences with asterisks used similarly to indicate significance versus MS1 cells.

## Results

### Adiponectin oligomers are large circulatory proteins

**Figure 1:**
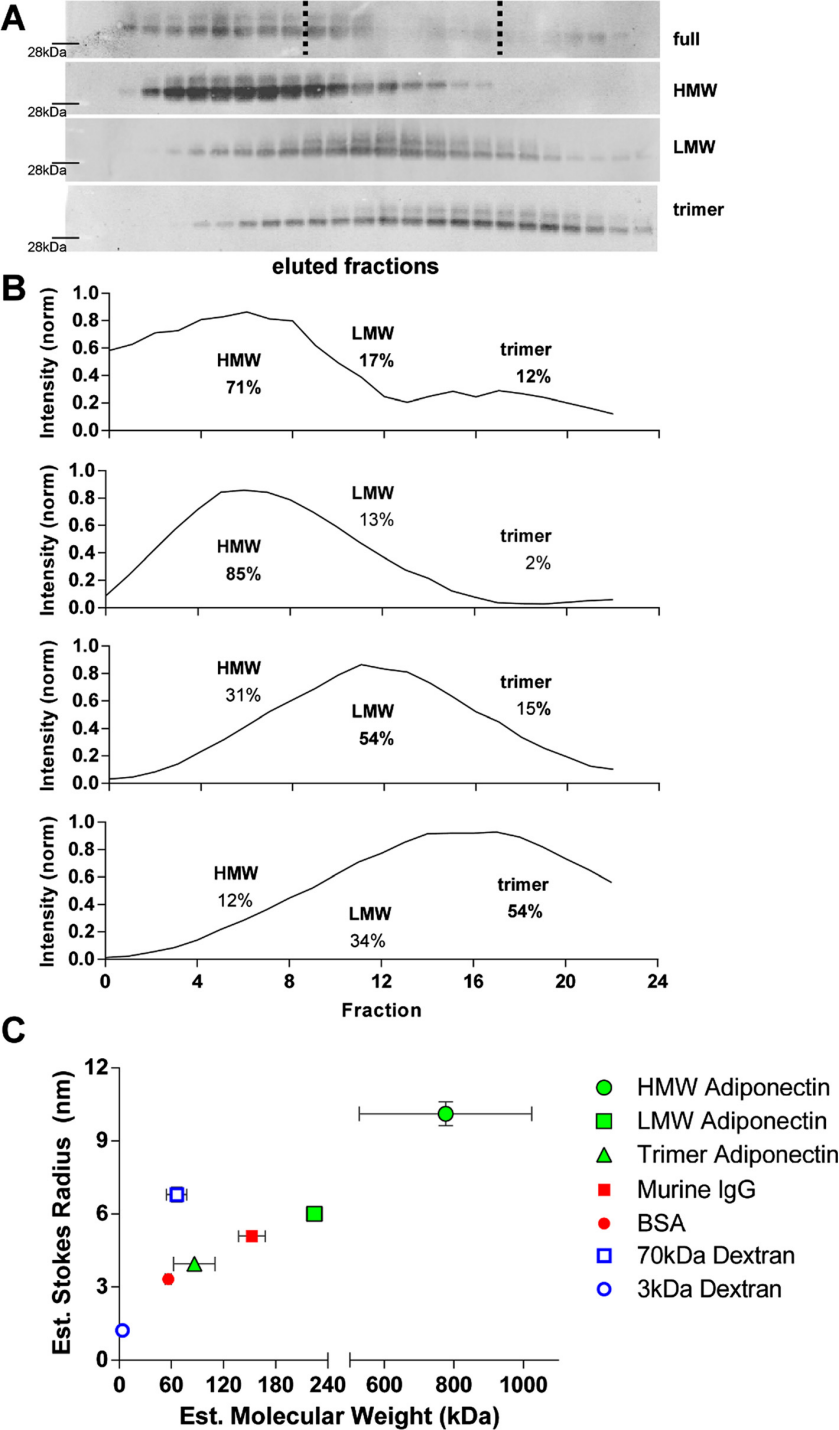
**Production and characterization of murine adiponectin oligomers. A)** Western blot of gel fractionated adiponectin oligomers from a full-length preparation. The highest molecular weight form elute first (left to right). Fractions representing HMW, LMW, and trimer adiponectin were pooled for their respective purifications. Dotted lines mark typical fraction cut-offs for pooling. The band for 28 kDa molecular weight marker (from the Bio-Rad 2 color ladder) is noted. The less intense slower band labeled is N-glycosylated – both bands were included in intensity quantification. **B)** Quantitation of oligomer distribution in purified preparations used in experiments; data are presented as 0^th^ order, 2-neighbor smoothed curves for clarity. Percent distributions were calculated from raw intensity data with peaks separated by fraction intensity peak valleys of the full-length form. **C)** Dynamic light scattering measurements of purified adiponectin oligomers yield estimated Stokes radii and molecular weights. Murine IgGs, albumin, and linear known-length dextran molecules were measured for comparison. All of the proteins – including adiponectin – followed a globular protein relationship while the dextrans fit a linear molecule relationship.

Under normal physiological conditions, adiponectin exists in circulation as trimeric, hexameric (LMW), and high molecular weight (HMW) oligomers. Mammalian HEK 293 T cells were necessary to achieve “full-length” (referring to the presence of all oligomers) recombinant murine adiponectin. The fractional distribution of HMW, LMW and trimer in the average production was assessed by gel filtration FPLC and subsequent immunoblotting of collected fractions (Figure 1A). HMW adiponectin was the dominant oligomer (71%) found in the recombinant protein production (Figure 1B). Purified complexes were isolated by repeated collection and pooling of the representative species’ elution. The resultant purified, concentrated oligomer preparations were analyzed by the same gel filtration FPLC method and demonstrated purities of 85%, 54%, and 54%, respectively, for the HMW, LMW, and trimeric preps (Figure 1B).

Diffusive transport of adiponectin through solution is determined by each oligomer’s Stokes radius. The Stokes radii of purified adiponectin complexes were measured by dynamic light scattering (DLS) and compared to other common circulatory proteins as well as linear known-size dextran molecules. The estimated molecular weights of each known species by DLS agreed with the published molecular weights of each respective standard molecule (Figure 1C). Adiponectin oligomers exhibited a linear relationship of estimated Stokes radii to molecular weight that is characteristic of globular proteins. Trimeric adiponectin was measured to have a Stokes radius of 3.96 nm, just slightly greater than albumin (3.33 nm). LMW adiponectin was approximately 200 kDa or 6.01 nm, a size larger than that measured for murine IgGs (5.09 nm). The HMW adiponectin preparation was very large, exhibiting an estimated molecular weight of nearly 800 kDa. The broad molecular weight size range measured was likely from the presence of 12-, 18-, 24- and 36-mers generally described – and isolated – as the HMW complexes. As these HMW forms are, however, tightly bundled globular proteins with the physical appearance of a bunch of tightly-held balloons [28], their transport radius of 10.1 nm did not increase as varyingly as the molecular weights. These large Stokes radii should limit adiponectin flux across endothelium and are highly relevant to the systemic fate of adiponectin in circulation.

### Adiponectin transport across murine endothelial cells is actively limited

**Figure 2:**
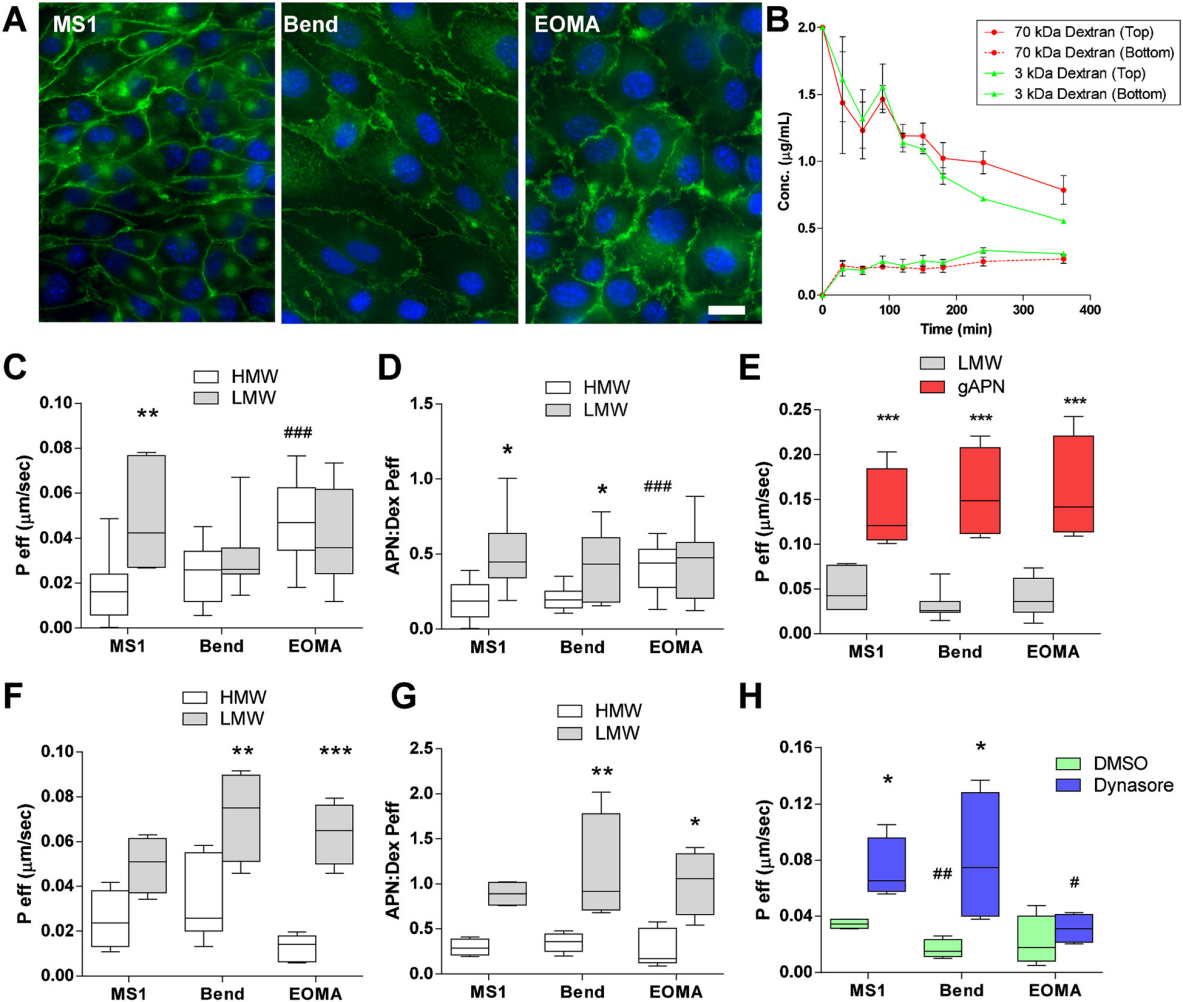
***In vitro*transwell assay to measure endothelial permeability to adiponectin complexes. A)** Murine endothelial cell lines MS1, bEnd.3 (Bend), and EOMA used in transwell assays to assess adiponectin flux exhibit different morphologies, but all demonstrate VE-Cadherin (green) positive junctions that inhibit paracellular large molecule transport (DAPI, blue; Bar?=?20 μm). **B)** 70 kDa and 3 kDa dextran flux across MS1-seeded transwells over time normalizes concentrations above and below the cells in approximately 8 hours. 6 hours was chosen for transport studies. **C)** Effective permeability (P_eff_) of LMW was significantly greater than HMW adiponectin across MS1 cells; HMW transport was greater across EOMA as compared to MS1 at 37°C. **D)** Ratios of adiponectin permeability to that of 3 kDa dextran demonstrated increased LMW permeability as compared to HMW in MS1 and Bend cells at 37°C. **E)** The P_eff_ of globular adiponectin (gAPN) is significantly greater than that of full-length adiponectin oligomers. **F)** The permeability of Bend and EOMA cells to LMW adiponectin was increased at 4°C, suggesting a loss of active barrier function for LMW adiponectin; HMW transport was unchanged. **G)** As a ratio to dextran, LMW adiponectin transport was greatly enhanced at 4°C.** H)** Inhibition of dynamin by Dynasore greatly enhanced full-length adiponectin transport in MS1 and Bend cells. No response was measured in EOMA cells. All analyses were by two-way (cell, treatment) ANOVA with Bonferroni posthoc analysis. *p < 0.05, **p < 0.01 and ***p < 0.001 compared to HMW. #*p* < 0.05, ##*p* < 0.01 and ###*p* < 0.001 compared to MS1 cells.

Model murine endothelial cell lines were used to quantify adiponectin oligomer transport across endothelial cell monolayers. MS1 cells were originally derived from pancreatic islets, Bend cells from the brain, and EOMA cells from a hemangioendothelioma – each cell line thus from tissues that exhibit differing molecular permeability. In culture, each cell line demonstrated clear endothelial cell cobblestone morphology at confluence and VE-cadherin expression at cell-cell junctions (Figure 2A). Each limited the transport of inert 3 kDa and 70 kDa dextran molecules when used in transwell permeability pilot assays for at least 8 hours (Figure 2B).

The permeability of each cell line to HMW and LMW adiponectin was quantified using transwell assay cultures at 37°C for 6 hours and measuring the transported species as described in the Methods. Quantified permeability of adiponectin across the model endothelial cells lines were subjected to a two-way analysis of variance having three types of cells and two types of oligomer (Figure 2C). Endothelial cell monolayer permeability to adiponectin was found to be both significantly dependent on cell type (*p*?=?0.013) and oligomer size (*p*?=?0.031). Bonferroni post-hoc analysis demonstrated a significant increase in LMW adiponectin transport over the HMW preparation across only MS1 cells (*p* < 0.01). Transport of HMW adiponectin across EOMA cells was significantly greater than MS1 or Bend cells (*p* < 0.01). Upon comparing the ratios of adiponectin permeability to those of a 70 kDa dextran tracer by the same analysis (Figure 2D), however, cell type was no longer significant (*p*?=?0.185), but the adiponectin oligomer effect was more pronounced (*p* < 0.001). The ratio of HMW adiponectin:dextran across MS1 cells was significantly less than LMW (*p* < 0.05). HMW adiponectin again exhibited increased transport across EOMA cells than MS1 or Bend cells (*p* < 0.05). It should be noted that because each adiponectin preparation did not consist 100% of a single oligomer size, the calculated P_eff_ may only be approximate. In all instances, adiponectin transport was far lower than dextran transport.

Commercially available globular adiponectin (gAPN) lacks the characteristic collagenous domain of endogenous adiponectin in circulation. To demonstrate the limitations of full-length adiponectin oligomers to move across the monolayer as opposed to gAPN, the permeability of gAPN across the endothelial cell lines were measured (Figure 2E). Compared to the permeability of our LMW adiponectin preps by two-way ANOVA, gAPN was significantly greater – more than a 3-fold increase – across all cells lines (each *p* < 0.001 by Bonferroni post-hoc analysis). The permeability was even greater than those of 70 kDa dextran, highlighting the physiologic limitations of the smaller recombinant form.

In experiments at 4°C, cell type was no longer significant (Figure 2F; *p*?=?0.070). Rather, oligomer size was highly determinant of endothelial monolayer permeability by two-way ANOVA (*p* < 0.001). Transport of LMW adiponectin was significantly greater than the HMW form across Bend and EOMA cells (*p* < 0.01 and *p* < 0.001, respectively). ANOVA identified that only transport of LMW adiponectin across Bend cells and HMW adiponectin across EOMA cells were significantly affected by temperature (both *p* < 0.001). LMW adiponectin permeability across all of the cells matched that of dextran (ratios near 1.0) suggested a loss of barrier function to adiponectin at 4°C.

Dynasore, a dynamin inhibitor, was used at 37°C to further explore active transport across the cells. Inhibition of dynamin had a greater effect (*p* < 0.001) than cell type (*p*?=?0.0685) by ANOVA on the transport of full-length adiponectin, with the most dramatic effect in Bend cells (*p* < 0.05). With both cold temperature and dynamin inhibition increasing adiponectin flux, the cells must actively regulate or limit its passage.

### Adiponectin action in endothelial cells

**Figure 3:**
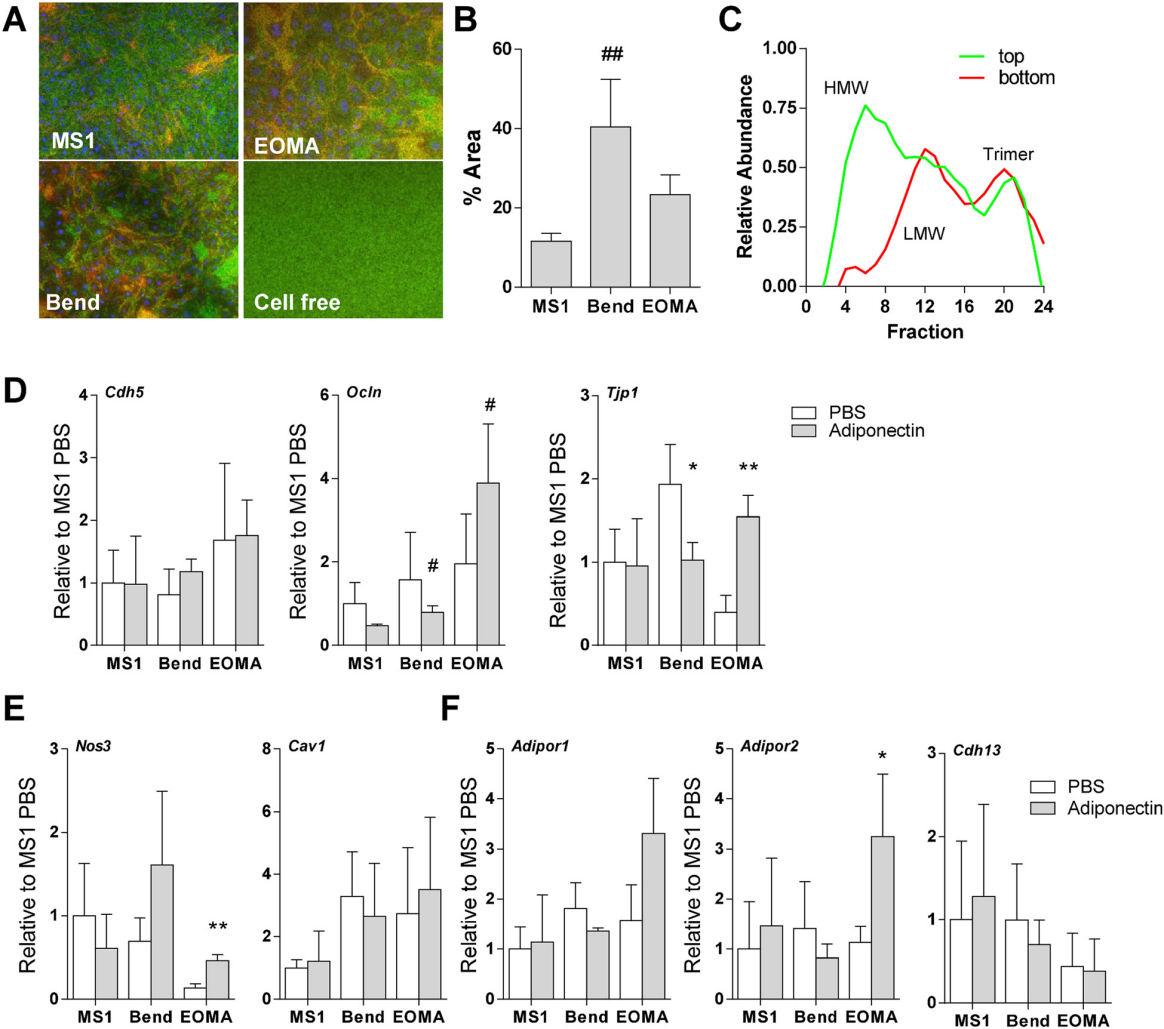
**Adiponectin uptake and modulation in endothelial cells. A)** A significant amount of IR800-labeled adiponectin was found attached or within endothelial cells following the transport experiments by confocal imaging of membranes (green?=?70 kDa dextran, red?=?adiponectin). Maximum projections are shown. **B)** Quantified adiponectin+?area on membranes; Bend cells bound or contained significantly more adiponectin than MS1 cells by ANOVA of image intensity analysis. **C)** After 6 hours of full-length adiponectin transport across MS1 cells, media from above the monolayer (top) and below the cells and membrane (bottom) was fractionated by gel filtration FPLC. Limited HMW was found to have crossed the cells. 3 samples were pooled for fractionation. **D)** No change in *Cdh5* gene expression was noted in the cells with adiponectin. Bend and EOMA cells had increased *Ocln* expression as compared to MS1 cells with adiponectin. These cells also increased *Tjp1* expression with adiponectin. **E)***Nos3* was regulated by adiponectin in EOMA cells. No changes were measured in *Cav1*. **(F)** Adiponectin receptors 1 and 2, respectively, were not regulated at the mRNA level by adiponectin treatment except for *Adipor2* in EOMA cells. *Cadh13* expression was not regulated by adiponectin in the cells. Gene comparisons were made by two-tailed student’s t-test with unequal variance. *p < 0.05 and **p < 0.01 with adiponectin. #*p* < 0.05 compared to MS1 cells.

Confocal microscopy of full-length IR800-labeled adiponectin revealed adiponectin to be localized to endothelial cell-cell junctions in each of the cell line monolayers on transwell membranes (Figure 3C). There was also substantial adiponectin within the cell layer, further indicating active transport of adiponectin across the endothelial monolayers. We measured the amount of bound or intracellular adiponectin after 6 hours of incubation with full-length adiponectin by image analysis of confocal maximum projections (Figure 3A). Each cell line held a significant quantity of adiponectin, with bEnd cells having significantly greater than MS1 cells (*p* < 0.01) by one-way ANOVA (Figure 3B). Media from atop a monolayer of MS1 cells and below the transwell membrane after 6 hours were sampled and fractionated by gel filtration FPLC (Figure 3C). All 3 oligomer sizes were transported across the cells as they were each detected in the bottom media. The adiponectin distribution atop the transwell was predominantly HMW, however, confirming the increased transport of the smaller forms – and exclusion of the larger oligomer – as measured with purified preparations (Figure 2C) across MS1 cells.

Adiponectin has been shown to regulate the expression of adhesion molecules in endothelial cells and thus may directly regulate transendothelial fluid transport. RNA extracted from transwell cultures after 6 hours in the presence or absence of recombinant full-length murine adiponectin demonstrated no marked changes in the RNA expression of the cell-cell junction proteins VE-cadherin (*Cdh5*) or occludin-1 (*Ocln*), but induced a significant decrease in *Tjp1* RNA for the junctional protein ZO-1 in Bend cells (*p*?=?0.040) and increase in EOMA cells (*p*?=?0.004; Figure 3D). *Ocln* levels in Bend and EOMA cells were significantly higher with adiponectin than in MS1 cells (*p*?=?0.031 and *p*?=?0.014, respectively). Expression levels of mRNA for *Enos* (Figure 3E), which may regulate vascular tone through nitric oxide *in vivo*, was significantly increased in EOMA cells (*p*?=?0.003). Caveolae are necessary for the active transendothelial transport of albumin and other proteins; its mRNA expression was not altered with adiponectin in any of the cells (*Cav1*; Figure 3E). Though it is unclear what role the adiponectin receptors play in cellular uptake of adiponectin, each of the endothelial cell lines was found to express *AdipoR1*, *AdipoR2*, and T-Cadherin (*Cdh13*). Expression of the receptors was not significantly regulated by exposure to adiponectin in the individual cell lines, with the exception of increased *Adipor2* mRNA in EOMA cells (*p*?=?0.046; Figure 3F). Even at basal metabolic conditions, adiponectin did exhibit some alteration in junctional molecule mRNA expression and some potential changes in nitric oxide production or adiponectin receptor expression, suggesting that these factors that regulate endothelial physiology may be further regulated in metabolic dysfunction.

### Adiponectin tissue uptake from circulation is dependent on vasoregulation and transport

**Figure 4:**
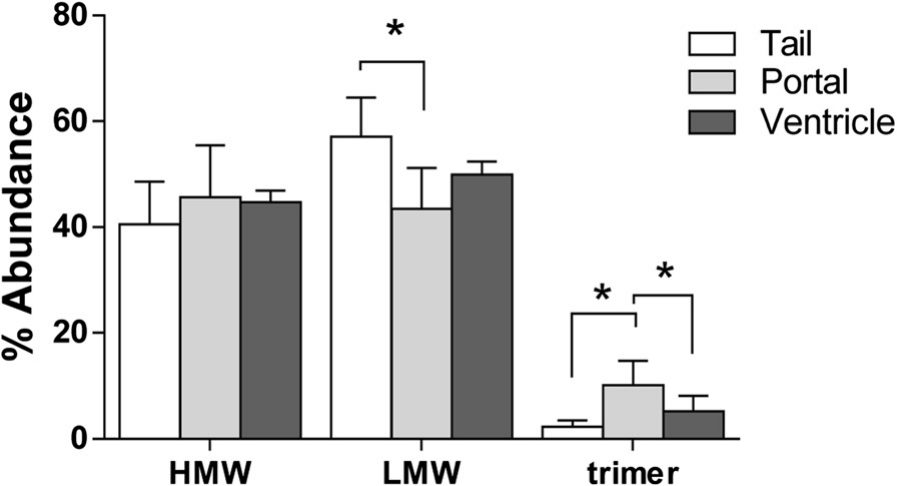
**Adiponectin oligomer distribution from different venous collections.** Sera collected from the tail vein, portal vein, and left ventricle of 5 mice under anesthesia was gel fractionated by FPLC and oligomer distributions quantified. The tail vein had significantly more LMW and less trimeric adiponectin than the portal circulation. Adiponectin trimers were also reduced at the heart compared to portal vein. Percent abundance was calculated as the summed intensity of peaks by immunoblot over the total intensity of the 24 eluted fractions; analysis by two-tailed student’s t-test with unequal variance. *p < 0.05 as indicated.

We recently demonstrated that the clearance of infused adiponectin from circulation was dependent on oligomer-size [18]. Having quantified adiponectin transport across endothelial monolayers and found significant differences between cell types and oligomer size, we sought to determine if different tissues preferentially take up adiponectin oligomers. Gel fractionation and quantification of endogenous adiponectin complex distribution in sera collected from the tail vein, portal vein, and left ventricle demonstrated significant differences in the relative oligomer abundance of LMW and trimer species (Figure 4). One-way ANOVA identified significant differences between the tail and portal circulation (*p* < 0.05) and significantly less trimer at the left ventricle than in portal sera (*p* < 0.05). This suggests that adiponectin complex distribution changes by location and likely reflects the differential clearance of the adiponectin complexes across the endothelia in different tissues. While supportive, we cannot rule out that adipose tissues from different anatomical regions contribute differential complements of adiponectin complexes to the circulating pool.

**Figure 5:**
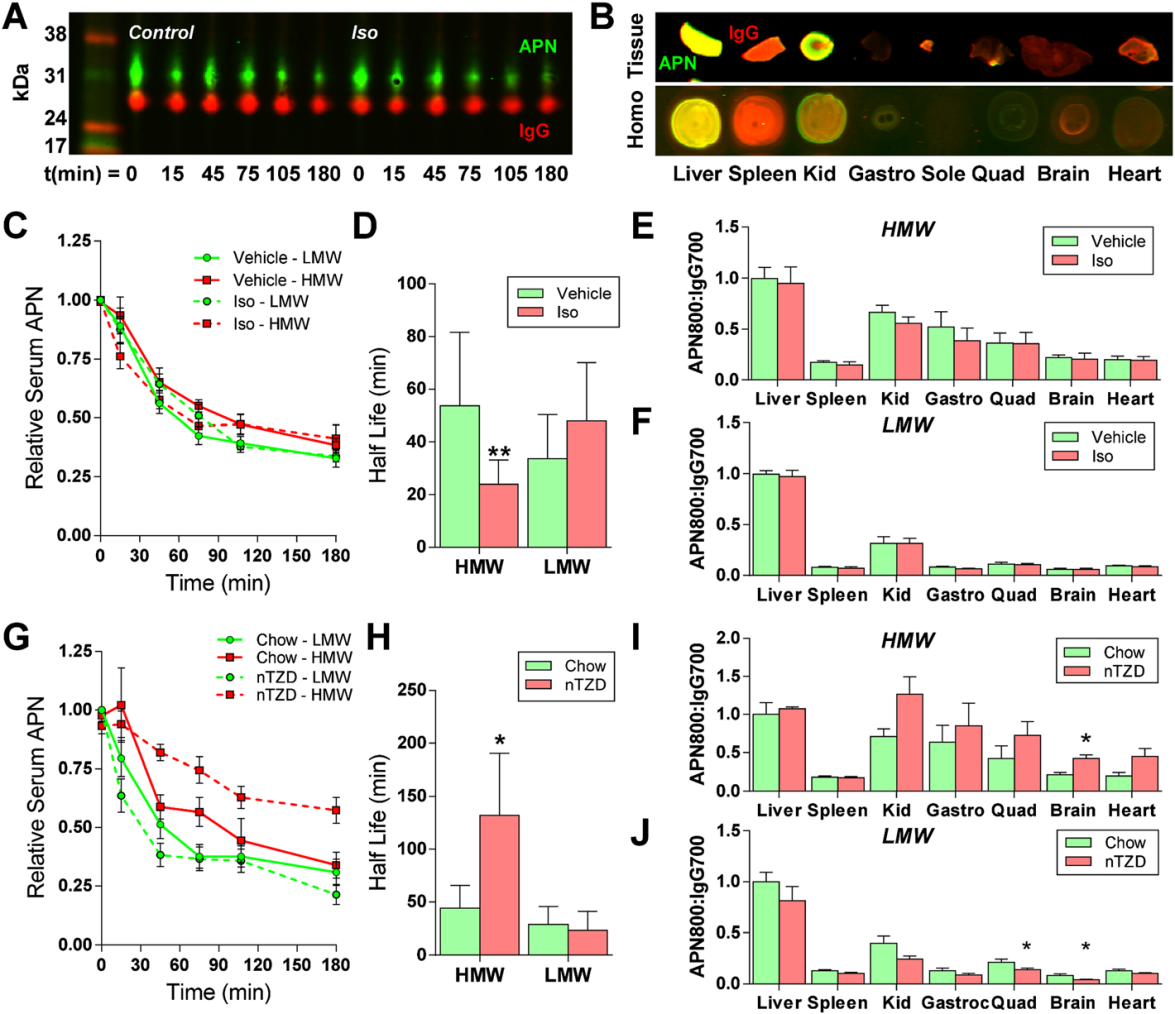
**Tracking labeled adiponectin oligomer circulatory clearance and tissue uptake over time. A)** Representative IR800-labeled adiponectin (green) and IR700-labeled IgG (red) over time from sera separated by gel electrophoresis; Iso: isosorbide dinitrate treatment. **B)** Three hours after injection, and following PBS perfusion, whole tissue was scanned to assess potential changes in adiponectin (green) localization and tissue homogenates, normalized for total protein concentration, were measured by dot blot quantification. Data are presented as adiponectin:IgG (red) ratio. **C)** Adiponectin:IgG ratios in sera over time following HMW (red lines) adiponectin or LMW (green lines) injection following acute vehicle (solid lines) and isosorbide dinitrate (Iso; dotted lines) administration. **D)** The half-life of HMW adiponectin was significantly reduced with iso treatment. **E)** Adiponectin:IgG ratios in tissues 3 hours following HMW adiponectin injection and normalized to liver values show no differences. **F)** Similarly, no differences were noted for LMW tissue uptake with isosorbide dinitrate. **G)** Adiponectin:IgG ratios in sera over time following HMW (red lines) adiponectin or LMW (green lines) injection into chow fed and PPARγ agonist (nTZD) diet fed (dotted lines) mice showed a delay in HMW clearance. **H)** Quantified half-lives demonstrated a significant decrease in HMW adiponectin clearance from circulation. **I)** nTZD treatment increased HMW adiponectin uptake in mouse brains. **J)** Conversely, nTZD-treated brains had significantly less LMW adiponectin uptake. Quadriceps also contained less adiponectin with the PPARγ agonist. Half-lives quantified by non-linear regression (decay) analysis. All values compared by two-tailed student’s t-test with unequal variance. *p < 0.05 between treatment and control.

To further quantify changes in circulating adiponectin *in vivo*, we assessed the circulatory half-life and tissue uptake of exogenously delivered IR800-labeled adiponectin when endothelial barrier function was pharmacologically-modulated. Following co-injection with an IR700-labeled mouse IgG, sera were sampled over 180 minutes. Collected sera were separated by gel electrophoresis to quantify adiponectin:IgG ratios and half-lives (Figure 5A). Perfused tissues were excised and the homogenate was similarly quantified by dot blot to assess tissue-specific uptake relative to IgG (Figure 5B).

Acute oral treatment with isosorbide dinitrate to induce vasodilation did not have a dramatic change on the circulatory clearance of either HMW or LMW adiponectin (Figure 5C). Calculated half-lives from this clearance data by non-linear regression analysis identified a significant reduction in the half life of HMW adiponectin, from 53.7 to 24.0 minutes, with nitroglycerine (*p*?=?0.001; Figure 5D). No significant changes were quantified in LMW adiponectin half life. Quantification of labeled adiponectin uptake into tissues demonstrated uptake primarily into liver and kidney with no significant differences detected between vehicle and isosorbide dinitrate treatment for HMW adiponectin (Figure 5E). Uptake of LMW adiponectin, regardless of treatment, was lower than HMW into most tissues as compared to the mouse livers (Figure 5F). Isosorbide dinitrate treatment did not significantly alter uptake of LMW adiponectin in the tissues surveyed.

PPARγ agonists are potent in restoring insulin sensitivity but have been limited clinically due to the changes in peripheral water balance and endothelial permeability with chronic use [29,30]. We utilized a 1-week nTZD treatment to determine if these conditions improved adiponectin flux into tissues. A marked retention of HMW adiponectin was noted with PPARγ agonism (Figure 5G). Indeed, the quantified half life was significantly extended from 44.4 to 132.1 minutes (*p*?=?0.026; Figure 5H). No equivalent change was measured in LMW adiponectin, suggesting that HMW transport may be actively modulated by PPARγ agonist treatment or that differential mechanisms exist for transport of HMW versus LMW forms. nTZD treatment also generally increased tissue uptake of HMW adiponectin as compared to chow diet, with significant elevations being identified in the brain (*p*?=?0.015; Figure 5I). The brain surprisingly exhibited reduced uptake of LMW adiponectin (*p*?=?0.023) upon nTZD treatment (Figure 5J). This dichotomous response may be regulated by the yet unknown changes in the adiponectin transport mechanism with PPARγ agonism identified in the half life calculation.

Caveolin-1 knockout mice have increased endothelial permeability due to increased paracellular albumin flux across the endothelium [25]. Surprisingly, this had no effect on adiponectin clearance from circulation (Additional file 1: Figure S1A). Neither HMW nor LMW oligomers demonstrated statistically different changes in circulation time with genotype (Additional file 1: Figure S1B) suggesting that adiponectin and albumin are transported differently across endothelium. HMW tissue uptake was significantly increased in the livers of caveolin-1 knockout mice (*p*?=?0.023; Additional file 1: Figure S1C), but in no other tissues measured. No changes in tissue distribution of LMW adiponectin were measured (Additional file 1: Figure S1D). While few significant differences were measured in half life or tissue uptake under the three conditions tested, these data demonstrate that adiponectin transport is certainly affected by changes in endothelial permeability and suggest that tackling dysfunctional endothelium pharmacologically may yield even greater changes in transendothelial flux.

### Increasing adiponectin action in skeletal muscle through exercise

**Figure 6:**
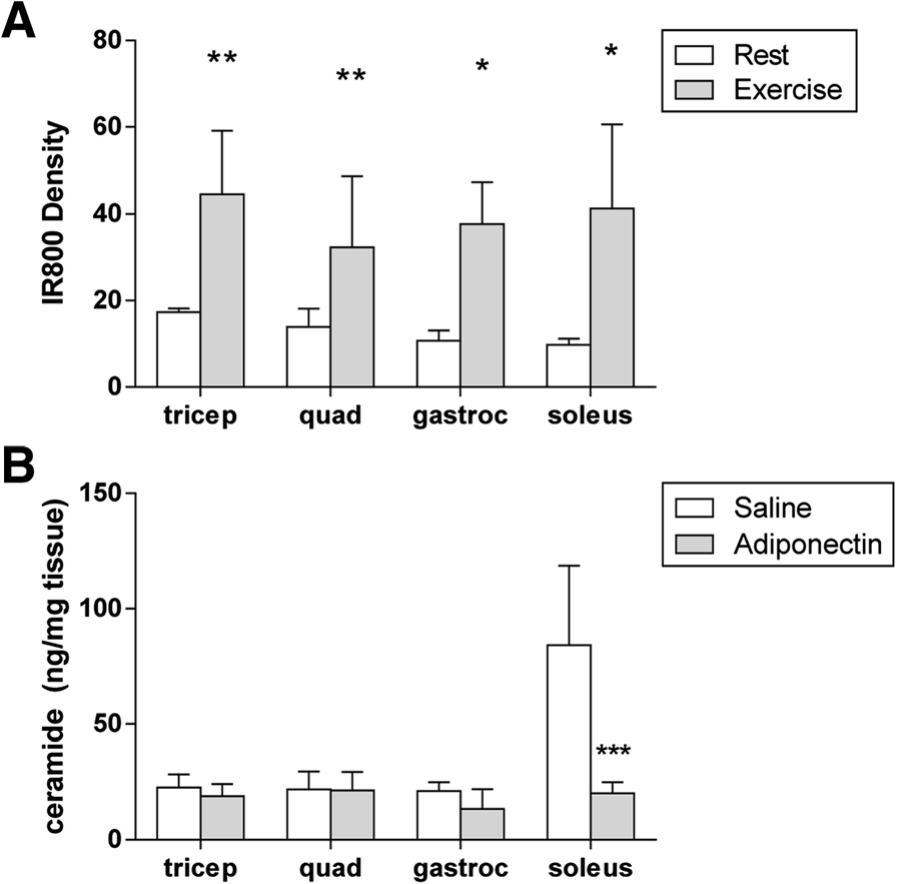
**HMW adiponectin uptake and function in skeletal muscle with exercise. A)** Significantly greater concentrations of IR800-labeled HMW adiponectin were measured in the tricep, quadricep (quad), gastrocnemius (gastroc) and soleus muscles of mice that had run for 2 hours following tail vein infusion as compared to rested mice. Density calculated by direct tissue scan. **B)** Total tissue ceramide content (C16:0-C24:0) of oxidative muscles was significantly reduced by adiponectin with the greatest effect being observed in isolated soleus muscle. *p < 0.05, **p < 0.01 and ***p < 0.001 as compared to controls by two-tailed student’s t-test with unequal variance.

According to a vast catalogue of literature, regulating skeletal muscle metabolism is a key action of adiponectin in insulin sensitization [31] and this response may be driven by adiponectin’s ability to lower cellular ceramide levels [27]. While our data have identified significant transport barriers of adiponectin across endothelium *in vitro*, our *in vivo* studies have demonstrated minimal changes in adiponectin uptake into skeletal muscle (Figure 5). Exercise is a condition with increased skeletal muscle blood flow, metabolism and ceramide content. To test whether the increasing blood flow to skeletal muscle might demonstrate increased adiponectin action there, IR800-labeled HMW adiponectin was administered to exercise-trained mice immediately prior to a two-hour run. At sacrifice, the tissue intensity of adiponectin label was significantly increased in tricep (*p*?=?0.006), quadricep (*p*?=?0.042), gastrocnemius (*p* < 0.001) and soleus (*p*?=?0.010) muscles (Figure 6A). Presence does not demonstrate function, so the total tissue ceramide content was measured by LC/MS. Quantified ceramide levels were significantly decreased with adiponectin treatment in soleus (*p*?=?0.033) and a trend towards reduction in gastrocnemius muscle (*p*?=?0.114) was measured. These oxidative muscles generate the most ceramide species, are highly responsive to adiponectin’s ceramidase induction and benefit the most from enhanced adiponectin uptake with exercise.

## Discussion

Here we report quantified transport sizes of adiponectin oligomers and demonstrate the endothelium to be a potent barrier to adiponectin transport. Numerous examples of changes in endothelial permeability to fluids and proteins have been noted with changes in metabolic state. The differential uptake of critical regulatory hormones – notably insulin [17,32] – and the active binding and transendothelial transport of fatty acids [33] to skeletal muscle is highly endothelial ‘health’ dependent. The size of adiponectin oligomers makes adiponectin a prime protein candidate for tight transport control. Indeed, of all proteins secreted by adipose tissue, adiponectin was the outlier demonstrating marked partitioning into blood despite its size (as opposed to the passive enrichment of other adipokines in lymph) suggesting active and preferential uptake by blood endothelium [34]. Beyond adipocyte secretion, circulatory uptake and clearance may be mechanisms behind changes in the distribution of circulating adiponectin complex measured here locally and systemically in, for example, the caveolin-1 knockout mouse [20]. Our *in vitro* data demonstrate that adiponectin oligomers are actively transported across different endothelium with differing efficiency and may be excluded or very limited; PPARγ agonism may change this mechanism. Our data also demonstrate a strong size-dependent regulation of adiponectin uptake from circulation. *In vivo*, small perturbations in endothelial barrier function or vessel dilation (and concomitant changes in microvascular flow rates) may regulate local tissue uptake. Adiponectin itself may signal in this regulation of local endothelial and tissue metabolism [35], most interestingly through the emerging adiponectin-sphingolipid axis which plays a role in endothelial function [36,37].

Existing data supports a strong correlation between cardiovascular health and endothelial responsiveness. Serum nitric oxide levels are greatly reduced with obesity and T2DM, for example; and skeletal muscle nitric oxide levels scaled with serum adiponectin levels [38]. Adiponectin uptake into tissue was not altered greatly with pharmacologic treatments in healthy mice, but was significantly increased with exercise and demonstrated potent ceramidase activity in this setting. Exercise, which increases both blood flow to muscle as well as nutrient flux, and healthy vascular tone may be necessary for the reported HMW adiponectin efficacy in skeletal muscle [39]. The combined data suggest that endothelial dysfunction in the metabolic syndrome may inhibit adiponectin clearance and uptake, as previously measured [18], and afford a target for enhancing adiponectin clearance.

Our tissue distribution data demonstrated changes in both HMW and LMW adiponectin uptake in the brain. Based on the full tissue scans and regional permeability, we hypothesize that this is uptake into the hypothalamus: hypothalamic adiponectin signaling has been identified as a potential regulator of glucose homeostasis and energy balance [40-42] and signaling throughout the brain may regulate mood and other processes [43,44]. Sampling of cerebral spinal fluid, however, found only trimeric adiponectin [45] and suggested that some reduction of adiponectin complexes is necessary to adiponectin to access the brain. Our data found that PPARγ agonism increased circulating HMW adiponectin and increased brain tissue levels; a concentration gradient driven flux is unlikely given the amounts injected. Indeed, proteolytic action on adiponectin oligomers has been suggested to direct adiponectin activity [46,47]; regulation of proteolytic activity with metabolic state may control HMW adiponectin clearance. Globular adiponectin, which already lacks the collagenous domain cleaved by digestion, is a potent inducer of adiponectin effects in muscle cells [48,49] but may have limited physiologic relevance as it would bypass these regulated transport sequences when studied *in vivo*.

Flux of adiponectin from adipose tissue or from the circulation into target tissue may play a role in the composition of circulating adiponectin. Fractionation of adiponectin complexes has identified that circulating levels of HMW adiponectin, or the ratio of HMW to trimer, may be the best indicator of insulin sensitivity and metabolic flexibility [2,3,6,20]. While these conclusions remain valid, recent studies have identified populations and BMI levels where this correlation is more significant with fat mass than disease state [50,51]. Other pathologies, such as chronic kidney disease, have also broken this general correlation [4]. Endothelial health and transport, levels of adiponectin receptor expression and binding, or depot-specific production and uptake may dictate complex clearance, distribution and function in these populations and pathologies. The relationship between adiponectin and endothelial function and metabolic responses continues to emerge [52].

## Conclusions

Adiponectin bioavailability for target cells under metabolically challenging conditions is reduced due to reduced trans-endothelial transport and may represent a subtle target for improving insulin sensitivity. Understanding adiponectin transport into and across these cells holds important clues towards understanding the systemic physiological effects of this important adipocyte-derived secretory protein and may further reinforce adiponectin as a predominant hormonal link between adipose tissue health and cardiovascular disease.

## Abbreviations

HMW: High molecular weight; LMW: Low molecular weight; gAPN: Globular adiponectin; nTZD: Non-thiazolidinedione PPARγ agonist; PPARγ: Peroxisome proliferator-activated receptor gamma; RIPA: Radioimmunoprecipitation assay buffer; BCA: Bicinchoninic acid; DLS: Dynamic light scattering.

## Competing interests

The authors declare that they have no competing interests.

## Authors’ contributions

JMR designed, carried-out, analyzed, and interpreted the experiments and wrote the manuscript. NH, QAW, and WLH helped design and carry-out experiments. JYX assisted with *in vivo* experiments. PES designed experiments, assisted in data interpretation, and edited the manuscript. All authors read and approved the final manuscript.

## Authors’ information

Assistant instructor JMR studied bioengineering at the Écolé Polytechnique Fédérale de Lausanne focusing on microvascular physiology for his PhD. Professor PES is a leading expert in metabolism research and the adipokine adiponectin. Together they sought to take a quantitative examination of adiponectin oligomer transport and how the endothelium may be a barrier to adiponectin function.

## Supplementary Material

Additional file 1: Figure S1 Tracking labeled adiponectin oligomer circulatory clearance and tissue uptake over time in wild type and *Cav1* knockout mice (Cav-/-). A) Adiponectin:IgG ratios in sera over time following HMW (red lines) adiponectin or LMW (green lines) injection into wildtype (WT; solid lines) and caveolin-1 knockout mice (Cav; dotted lines). B) Lack of caveolar trafficking had no effect on either adiponectin complex half life. C) HMW adiponectin tissue distribution was unchanged in Cav -/- mice. D) LMW adiponectin concentration was increased in the brains of Cav-1 KO mice. Analyses by two-tailed student’s t-test with unequal variance. * *p*<0.05. **Table S1**. Primer sequences used for quantitative PCR measures in murine endothelial cells. Click here for file
