# Supplementary material for: Differential transendothelial transport of adiponectin complexes
Source: Cardiovasc Diabetol. 2014 Feb 20;13:47. doi: 10.1186/1475-2840-13-47 (PMC3932731; doi:10.1186/1475-2840-13-47)
Supplement: Additional file 1: Figure S1 — Tracking labeled adiponectin oligomer circulatory clearance and tissue uptake over time in wild type and Cav1 knockout mice (Cav-/-). A) Adiponectin:IgG ratios in sera over time following HMW (red lines) adiponectin or LMW (green lines) injection into wildtype (WT; solid lines) and caveolin-1 knockout mice (Cav; dotted lines). B) Lack of caveolar trafficking had no effect on either adiponectin complex half life. C) HMW adiponectin tissue distribution was unchanged in Cav -/- mice. D) LMW adiponectin concentration was increased in the brains of Cav-1 KO mice. Analyses by two-tailed student’s t-test with unequal variance. * p<0.05. Table S1. Primer sequences used for quantitative PCR measures in murine endothelial cells. [file 1475-2840-13-47-S1.pdf]

# Figure S1

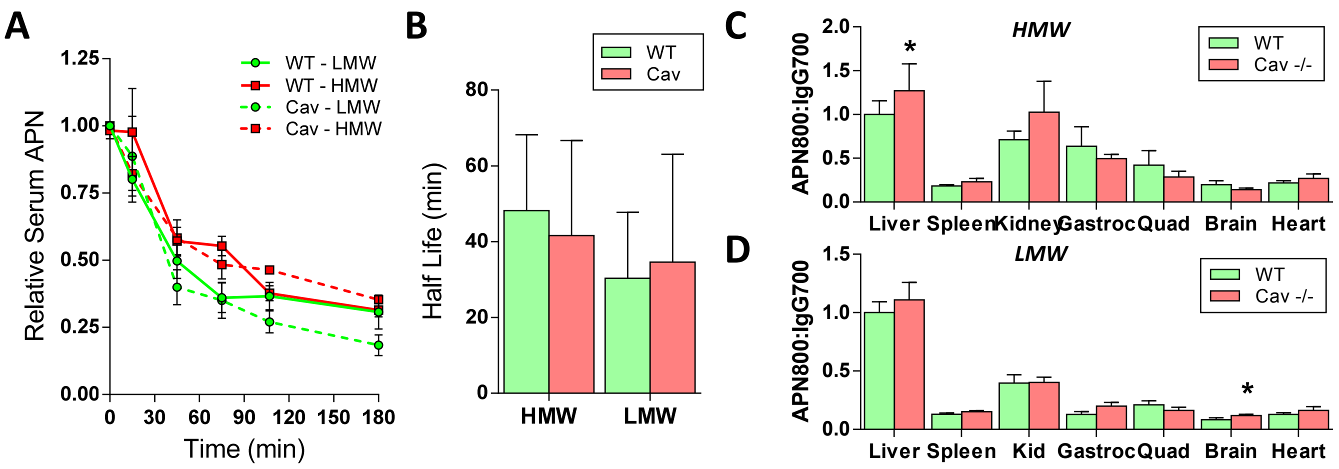


**Supplemental Figure 1:** Tracking labeled adiponectin oligomer circulatory clearance and tissue uptake over time in wild type and *Cav1* knockout mice (Cav-/-). A) Adiponectin:IgG ratios in sera over time following HMW (red lines) adiponectin or LMW (green lines) injection into wildtype (WT) and caveolin-1 knockout mice (Cav; dotted lines). B) Lack of caveolar trafficking had no effect on either adiponectin complex half life. C) HMW adiponectin tissue distribution was unchanged in Cav -/- mice. D) LMW adiponectin concentration was increased in the brains of Cav-1 KO mice. Analyses by two-tailed student's t-test with unequal variance. * P<0.05.

# Table S1

| Gene | Forward | Reverse |
|---|---|---|
| *Cdh5* | TCCTCTGCATCCTCACCATCACA | GTAAGTGACCAACTGCTCGTGAAT |
| *Ocln* | CCTTCTGCTTCATCGCTTCC | AGCGCTGACTATGATCACGA |
| *Tjp1* | TCAGAGCCCTCCGATCATTC | GCTTTGGGTGGATGATCGTC |
| *Nos3* | TTCCTGGACATCACTTCCCC | CTTCCATTCTTCGTAGCGCC |
| *Cav1* | CCAAGCATCTCAACGACGAC | CTTCCAGATGCCGTCGAAAC |
| *Adipor1* | ACGTTGGAGAGTCATCCCGTAT | CTCTGTGTGGATGCGGAAGAT |
| *Adipor2* | TCCCAGGAAGATGAAGGGTTTAT | TTCCATTCGTTCCATAGCATGA |
| *Cdh13* | CATCGAAGCTCAAGATATGG | GATTTCCATTGATGATGGTG |

**Supplementary Table 1:** Primer sequences used for quantitative PCR measures in murine endothelial cells.
